# Immunoglobulin-like transcript 2 as an impaired anti-tumor cytotoxicity marker of natural killer cells in patients with hepatocellular carcinoma

**DOI:** 10.3389/fimmu.2024.1389411

**Published:** 2024-04-04

**Authors:** Toshihiro Sakata, Sachiyo Yoshio, Taiji Yamazoe, Taizo Mori, Eiji Kakazu, Yoshihiko Aoki, Nobuyoshi Aoyanagi, Toru Okamoto, Takanori Ito, Hidenori Toyoda, Takumi Kawaguchi, Yoshihiro Ono, Yu Takahashi, Akinobu Taketomi, Tatsuya Kanto

**Affiliations:** ^1^ Department of Liver Diseases, The Research Center for Hepatitis and Immunology, National Center for Global Health and Medicine, Chiba, Japan; ^2^ Department of Gastoenterological Surgery I, Hokkaido University Graduate School of Medicine, Hokkaido, Japan; ^3^ Kohnodai Hospital, National Center for Global Health and Medicine, Chiba, Japan; ^4^ Department of Microbiology, Juntendo University School of Medicine, Tokyo, Japan; ^5^ Division of Gastroenterology and Hepatology, Nagoya University Graduate School of Medicine, Aichi, Japan; ^6^ Department of Gastroenterology, Ogaki Municipal Hospital, Gifu, Japan; ^7^ Division of Gastroenterology, Department of Medicine, Kurume University School of Medicine, Fukuoka, Japan; ^8^ Division of Hepatobiliary and Pancreatic Surgery, Cancer Institute Hospital, Japanese Foundation for Cancer Research, Tokyo, Japan

**Keywords:** NK cells, ILT2, HLA-G, HCC, MIF, ADCC

## Abstract

**Introduction:**

Natural killer (NK) cells play a pivotal role in immune surveillance in the liver. We aimed to identify potential targets for NK cell-mediated immune intervention by revealing the functional molecules on NK cells in HCC patients.

**Methods:**

To evaluate the impact of aging on NK cell phenotypes, we examined NK cells from healthy volunteers (HVs) of various ages. Because ILT2 expression on CD56^dim^ NK cells increased with increasing age, we enrolled age-matched HCC patients and HVs. We determined the NK cell phenotypes in blood mononuclear cells (PBMCs) and intrahepatic lymphocytes (IHLs) from cancerous and non-cancerous tissues. We evaluated cytotoxicity and antibody-dependent cellular cytotoxicity (ADCC) of NK cells in vitro.

**Results:**

ILT2-positive CD56^dim^ NK cells in PBMCs were increased in HCC patients compared with HVs. In HCC patients, ILT2-positive CD56^dim^ NK cells were increased in cancerous IHLs compared with non-cancerous IHLs and PBMCs. We examined the impact of macrophage migration inhibitory factor (MIF) on ILT2 expression in co-cultures of HCC cells and NK cells. The enhanced expression of ILT2 on CD56^dim^ NK cells from HCC patients was inhibited by masking antibodies against MIF and CXCR4. ILT2-positive CD56^dim^ NK cells exhibited lower capacities for cytotoxicity and ADCC than ILT2-negative cells, which were partially restored by ILT2 blockade.

**Conclusions:**

In HCC patients, ILT2 is a signature molecule for cancerous CD56^dim^ NK cells with impaired cytolytic capacity. The MIF-CXCR4 interaction is associated with ILT2 induction on CD56^dim^ NK cells and ILT2 serves as a target for functional NK cell restoration.

## Graphical Abstract

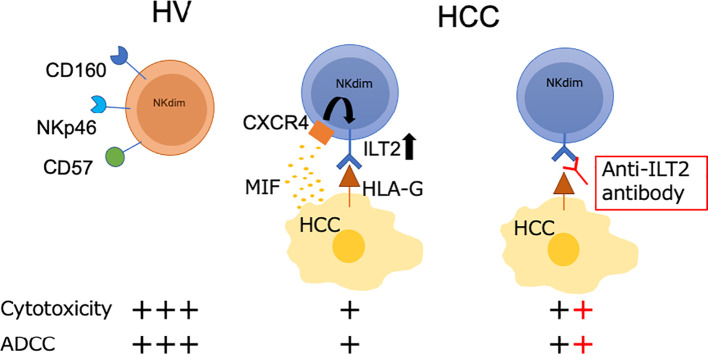

## Introduction

HCC is the most common form of liver cancer and was the third most common cause of cancer death in 2020 (1, 2). Treatments for advanced HCC have improved considerably over the last few years. Combination therapies including immune checkpoint inhibitors (ICIs) have become the standard treatment for patients with unresectable HCC (3). However, the response rates to systemic therapy remain unsatisfactory. Consequently, identification of new therapeutic targets for immunological intervention is required to improve the prognosis of patients with advanced HCC. Natural killer (NK) cells play a critical role in regulating immune responses against tumors (4, 5) and are involved in the responsiveness of patients to ICI therapy (6). A reduction in intratumor CD56^+^ NK cells was found to be correlated with poor prognosis in HCC patients (7). Thus, a profound analysis of the phenotypes and functions of NK cells in HCC patients may provide useful insights into possible immunomodulatory strategies.

Human NK cells are classified into two subsets according to their expression of CD56 and CD16 (Fc-gamma receptor IIIa [FcγRIIIa]). CD56^dim^CD16^+^ NK cells exhibit high cytotoxic activity, while CD56^bright^CD16^−^ NK cells are potent cytokine producers. The ratios of NK cells and their subsets are completely distinct between the peripheral and inner regions of the liver, being approximately 10% in peripheral blood mononuclear cells (PBMCs) and 30%–50% in intrahepatic lymphocytes (IHLs) (4, 5). In healthy individuals, CD56^dim^CD16^+^ NK cells account for 90% of the peripheral NK cell population. In a previous study on HCC patients, we found that CD56^dim^ NK cells were dominant in the liver, and that the frequency of intratumor CD56^dim^ NK cells was reduced compared with intrahepatic non-tumor CD56^dim^ NK cells (5). Therefore, it is arguably necessary to analyze intrahepatic and intratumor NK cells in patients with HCC.

The capacity of NK cells is regulated by the balance of activating and inhibitory receptors in the tumor microenvironment (TME). Aging is a biological process associated with dynamic editing of the immune system, often accompanied by gradual impairment of immune surveillance against tumors (6). Several lines of evidence have shown that HCC and aging have negative impacts on NK cell functions (4, 6, 8). However, it remains unclear how aging and presence of HCC affect the expression of function-related molecules on NK cells.

In this study, we aimed to identify potential targets for NK cell-mediated immune intervention. To this end, we comprehensively examined the phenotypes and functions of NK cells in patients with HCC, in relation to the age of the patients and the localizations of NK cells. We found that ILT2^+^NKp46^−^CD56^dim^ NK cells had impaired cytolytic and antibody-dependent cellular cytotoxicity (ADCC) capacities in HCC patients, and that these capacities were restored by anti-ILT2 antibody treatment. We further found that macrophage migration inhibitory factor (MIF) was partially involved in the induction of ILT2 on NK cells.

## Materials and methods

### Subjects

We enrolled 17 patients with no or mild fibrosis (fibrosis [F] stage 0, 1, or 2; *n*=6) or advanced fibrosis (F stage 3 or 4; *n*=11) who underwent liver resection for HCC at Kohnodai Hospital or the Cancer Institute Hospital of the Japanese Foundation for Cancer Research between May 2018 and December 2020 (**Supplementary Table 1**). As controls, we enrolled 42 healthy volunteers (HVs) who ranged in age from 21 to 82 years, had no apparent history of liver diseases or malignancies, and were negative for HBsAg, HIV antigen, anti-HIV antibodies, and anti-HCV antibodies. Written informed consent was obtained from all subjects at enrollment. The study conformed to the ethical guidelines of the 1975 Declaration of Helsinki and the ethical guidelines for human clinical research established by the Japanese Ministry of Health, Labour and Welfare. The study protocol was approved by the ethics committees of the National Center for Global Health and Medicine (NCGM-A-000275-01) and the Cancer Institute Hospital of the Japanese Foundation for Cancer Research (2017-GA-1118).

### Cell lines

The K562 cell line (#JCRB0019; JCRB) was cultured in RPMI-1640 medium (Thermo Fisher Scientific, Waltham, MA) supplemented with 10% heat-inactivated fetal bovine serum (FBS) (GE Healthcare, Chicago, IL). The Daudi cell line (#JCRB9071; JCRB) was cultured in RPMI-1640 medium supplemented with 20% heat-inactivated fetal FBS. Huh7and PLC-PRF5 were kindly gifted by Prof. Okamoto T., and HLE cells were by Prof. Kawaguchi T. These cell lines were cultured in DMEM (043-30085; Wako, Osaka, Japan) supplemented with 10% FBS, 1% penicillin-streptomycin (P4333; Sigma-Aldrich, St. Louis, MO), 1% MEM (M7145; Sigma-Aldrich), and 10 mM HEPES (Nacalai Tesque, Kyoto, Japan). All cells were incubated at 37°C in a humidified 5% CO_2_ incubator. All experiments were performed with mycoplasma-free cells.

### Isolation of peripheral and intrahepatic mononuclear cells

PBMCs were isolated by density gradient centrifugation on Ficoll-Paque (d=1.077; Nacalai Tesque). For isolation of IHLs, HCC tissues and adjacent normal liver tissues were promptly transported from the hospital to the laboratory on ice in RPMI-1640 medium containing 2 mM L-glutamine (Thermo Fisher Scientific), 25 mM HEPES (Nacalai Tesque), 10% fetal calf serum (FCS) (HyClone, Cytiva, Tokyo, Japan), and 100 U/mL penicillin/streptomycin (Nacalai Tesque) (Buffer 1). The liver tissues were washed twice with HBSS (Gibco) containing 2% FCS and 0.6% bovine serum albumin (Buffer 2), minced, and enzymatically digested with 50 µg/L DNase I (Promega, Madison, WI) and 500 mg/L collagenase IV (Nordmark Arzneimittel Gmbh & Co. KG, Uetersen, Germany) for 60 min at 37°C as reported previously (4). After the obtained cell suspension was filtered through a 40-μm cell strainer (Greiner), IHLs were isolated by density gradient centrifugation on Ficoll-Paque (d=1.077; Nacalai Tesque) and CD45^+^ IHLs were obtained using a MACS system (Miltenyi Biotec, Bergisch Gladbach, Germany). PBMCs and IHLs were harvested and stored at −150°C in Cell Banker solution (ZENOAQ Resource Co. Ltd., Fukushima, Japan). Following the density gradient centrifugation, the residual cells including hepatocytes and erythrocytes were resuspended with ACK lysis buffer (1.5M NH4Cl, 100mM KHCO3, and 0.5M EDTA adjusted to PH 7.2) to lyse the erythrocytes. After washing, the hepatocytes were isolated by centrifugation on a Percoll (GE Healthcare Bio-Sciences, Uppsala, Sweden) cushion (1.129 g/mL). For NK cell isolation, NK cells were purified by negative magnetic selection using an NK Cell Isolation Kit (Miltenyi Biotec). To obtain ILT2^+^CD56^dim^ NK cells, isolated NK cells were stained with a LIVE/DEAD™ Fixable Aqua Dead Cell Stain Kit (L34966; Invitrogen) and anti-CD3/CD14/CD19-APC-Cy7 (Biolegend, San Diego, CA), anti-CD56-V450 (BD Biosciences, San Jose, CA), anti-CD16-PE (BD Biosciences), anti-NKp46-APC (BD Biosciences), and anti-ILT2-PE-Cy7 (Biolegend) antibodies for 30 min at 4°C. The stained cells were sorted in a FACS Aria III cell sorter (BD Biosciences).

### Mass cytometry by time-of-flight (CyTOF)

**Figure 1:**
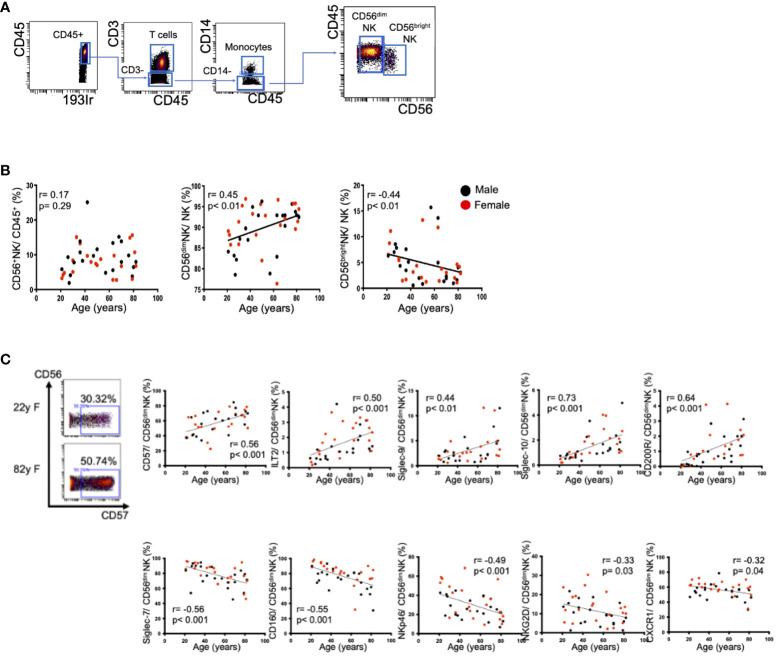
Changes in peripheral CD56^dim^ NK cells with aging. **(A)** Representative dot plots showing the gating strategy for CD56^dim^ NK cells (CD45^+^CD3^−^CD14^−^CD56^dim^) and CD56^bright^ NK cells (CD45^+^CD3^−^CD14^−^CD56^bright^). The arrows indicate the gating sequence. **(B)** Spearman’s correlations for peripheral CD56^+^ NK cells, CD56^dim^ NK cells, and CD56^bright^ NK cells in HVs with age. **(C)** Representative mass cytometry plots for CD56 and CD57 on peripheral CD56^dim^ NK cells from a younger HV and an older HV. The remaining panels show the Spearman’s correlations between age and the percentages of CD57, Siglec-7, CD160, NKp46, NKG2D, CXCR1, ILT2, Siglec-9, Siglec-10 and CD200R expression on peripheral CD56^dim^ NK cells in HVs. Black and red plots indicate data for men and women, respectively. The values of the Spearman’s correlation coefficients are indicated.

The CyTOF procedure employs metal isotope-conjugated antibodies that are distinguishable by mass in a time-of-flight mass spectrometer, thereby allowing simultaneous detection of a large number of markers without the spectral overlap limitations inherent to fluorophore-based flow cytometry. For analysis, the cells were thawed, treated with cisplatin (Fluidigm, San Francisco, CA) to identify live/dead cells, and incubated with metal-conjugated antibodies against surface membrane proteins (listed in **Supplementary Table 2**). The cells were fixed with 1.6% paraformaldehyde, labeled with an iridium-containing DNA intercalator to allow discrimination between singlets and doublets, and analyzed using a CyTOF mass cytometer (Helios, Fluidigm). The CyTOF signals were normalized using EQ Four Element Calibration Beads (201078; Fluidigm) in accordance with the manufacturer’s instructions. The data files were analyzed using Cytobank software (Cytobank Premium, Mountain View, CA). A total of 40,000 CD45^+^ leukocytes were analyzed per sample. The gating strategy used to identify NK cells (CD45^+^CD3^−^CD14^−^CD56^+^), T cells (CD45^+^CD3^+^), and monocytes (CD45^+^CD3^−^CD14^+^) is shown in **Figure 1A**. The data files generated from the CyTOF analysis were subjected to a dimension reduction process based on the viSNE algorithm, which allows multidimensional cytometry data to be presented in two dimensions while retaining the multidimensional data structure (9). For the viSNE analysis, a total of 14,000 NK cells from each donor were included and the data were clustered on the basis of the mean signal intensity (MSI).

### Flow cytometry

All cells were incubated with FcR blocking reagent (130-059-901; Miltenyi Biotec) for 10 min at 4°C before cell surface staining. For NK cell phenotyping, PBMCs and IHLs were stained with a LIVE/DEAD™ Fixable Aqua Dead Cell Stain Kit (L34966; Invitrogen) and anti-CD3/CD14/CD19-APC-Cy7 (Biolegend), anti-CD56-BUV737 (BD Biosciences), anti-CD16-BUV395 (BD Biosciences), anti-NKp46-BV421 (BD Biosciences), and anti-ILT2-PE-Cy7 (Biolegend) antibodies for 30 min at 4°C. For ILT2 ligand analysis, hepatocytes from HCC tissue (Ca) and adjacent non-cancerous liver tissue (NCa) were incubated with PE-HLA-G and isotype control. All samples were acquired on an LSRFortessa (BD Biosciences) with FACSDiva software and analyzed using FlowJo 10.8.1 software (BD Biosciences). The antibodies used for the flow cytometry analysis are listed in **Supplementary Table 3**.

### Cytotoxicity and ADCC assay

NK cell cytotoxicity and ADCC were analyzed by flow cytometry as described previously (10). Carboxyfluorescein succinimidyl ester (CFSE) (C34554; Invitrogen)-labeled K562 and Daudi cells were resuspended in RPMI-1640 medium supplemented with 10% FBS and placed in 96-well V-bottomed plates at 4×10^3^ cells/200 μL/well. Isolated NK cells (effector cells) were incubated in triplicate with CFSE-labeled K562 and Daudi cells (target cells) at 5:1 and 10:1 effector/target ratios, respectively, for 4 h at 37°C in a 5% CO_2_ incubator. K562 and Daudi cells expressed HLA-G (**Supplementary Figure 1**). sIL-2 (50 ng/mL) (200-02; Thermo Fisher Scientific) was added before the cytotoxicity assay. Rituximab (10 μg/mL) (HY-P9913; MedChemExpress, Monmouth Junction, NJ) was added before the ADCC assay. Before the functional recovery assay, isolated NK cells were incubated with an ILT2-blocking antibody (10 μg/mL) (333721; Biolegend) or isotype antibody (10 μg/mL) (401216; Biolegend) for 1 h. Pre-treated NK cells (effector cells) were incubated with CFSE-labeled K562 and Daudi cells (target cells) at 2.5:1 effector/target ratios for 4 h at 37°C in a 5% CO_2_ incubator. After 4 h of incubation, the target cells were lysed, stained with 7-AAD (559925; BD Biosciences) for 10 min, and analyzed in the LSRFortessa using FACSDiva software and FlowJo 10.8.1 software. Lysis was quantified by calculating the percentage of 7-AAD-positive cells among all CFSE-positive target cells. The specific cell lysis was calculated using the following formula: % specific lysis = (% lysis of sample cells – % lysis of control target cells)/(100% – % lysis of control target cells). The antibodies used for the flow cytometry analysis are listed in **Supplementary Table 3**.

### Chemokine and cytokine assays

Serum samples were prepared by centrifugation at 300×*g* for 15 min and stored at −80°C. A Bio-Plex Pro™ Human Chemokine Panel (40 plex) (Bio-Rad, Hercules, CA) was used in accordance with the manufacturer’s instructions. The complete panel screened for expression of the following chemokines: CCL1, CCL2, CCL3, CCL7, CCL8, CCL11, CCL13, CCL15, CCL17, CCL19, CCL20, CCL21, CCL22, CCL23, CCL24, CCL25, CCL26, CCL27, CX3CL1, CXCL1, CXCL2, CXCL5, CXCL6, CXCL8, CXCL9, CXCL10, CXCL11, CXCL12, CXCL13, CXCL16, GM-CSF, IFN-γ, IL-1β, IL-2, IL-4, IL-6, IL-10, IL-16, MIF, and TNF-α.

### Cell culture

Co-cultures of 5×10^4^ Huh7, PLC-PRF5, or HLE cells and 5×10^4^ magnetic cell-sorted NK cells from HVs were conducted with recombinant IL-15 1 ng/mL (247-ILB-005; R&D Systems, Minneapolis, MN) in a 24-well plate for 72 h. For co-cultures using transwell inserts, 5×10^4^ NK cells were cultured in the upper chamber with a 0.4-μm pore polyethylene terephthalate (#353495; Corning, Glendale, AZ). In other cultures, 5×10^4^ magnetic cell-sorted NK cells from HVs were cultured for 48 h in RPMI-1640 medium supplemented with 10% heat-inactivated FBS and recombinant human MIF (289-MF-01M/CF; R&D Systems) or 50% cell culture supernatants from HCC cell lines in the presence or absence of isotype control antibody, DMSO, blocking anti-MIF antibody (MAB289; R&D Systems), blocking anti-CD74 antibody (555612; BD Biosciences), CXCR2 antagonist (SB225002; MedChemExpress), or CXCR4 antagonist (WZ811; Selleckchem.com).

### Statistical analysis

Differences between two groups were evaluated by the Mann–Whitney U-test or Wilcoxon signed-rank test. Differences between more than two groups were evaluated by the Kruskal–Wallis test with Dunn’s multiple comparison test. Correlations were assessed using Spearman’s analysis. Statistical analyses and data visualization were performed using Prism software version 8 (Graph Pad, San Diego, CA).

## Results

### Impact of age on NK cells in healthy subjects

Aging is one of the important factors impacting the frequency and functions of immune cells. Initially, we evaluated the frequencies and expression levels of surface markers on peripheral NK cells in 42 HVs. We defined CD56^dim^ and CD56^bright^ NK cells as shown in **Figure 1A**. The frequency of CD56^dim^ peripheral NK (pNK) cells increased with age, while the frequency of CD56^bright^ NK cells decreased (**Figure 1B**). These tendencies did not differ by sex. The expression levels of CD57, a maturation marker, and four inhibitory receptors (ILT2, Siglec-9, Siglec-10, CD200R) were positively correlated with age (**Figure 1C**, **Supplementary Table 4**). In contrast, five activation markers (Siglec-7, CD160, NKp46, NKG2D, CXCR1) were negatively correlated with age (**Figure 1C**, **Supplementary Table 4**). Because CD56^dim^ NK cells are one of the major types of effector cells with cytolytic activity, we focused on the CD56^dim^ subset in the following experiments. Representative viSNE plots of pNK cells from a younger individual (22 years of age) and an older individual (82 years of age) revealed that CD57^high^Siglec-7^low^CD160^low^NKp46^low^CD56^dim^ NK cells were increased in the older individual and that the same cell population expressed immune checkpoint molecules such as PD-1, LAG-3, and TIGIT (**Supplementary Figure 2**). These results suggest that, in accordance with aging, NK cells in healthy individuals tended to decrease with enhanced expression of inhibitory receptors and decreased expression of activating receptors. These phenotypic alterations may be involved in potential NK cell dysfunction in older individuals. Thus, the impact of aging on NK cells requires consideration when comparative NK cell analyses are conducted between HCC patients and healthy subjects.

### Changes in NK cells in HCC patients

**Figure 2:**
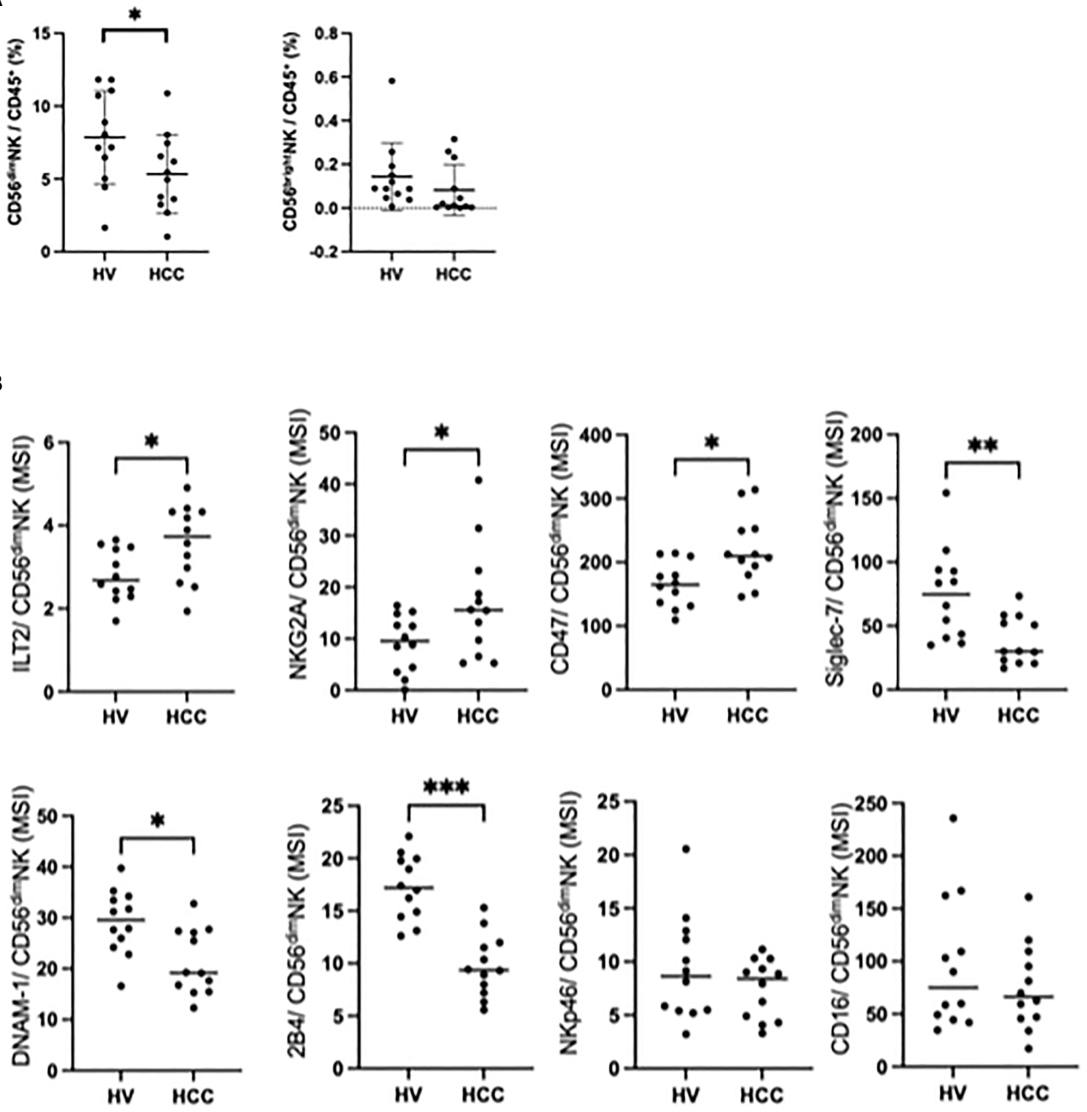
Changes in peripheral CD56^dim^ NK cells in HCC patients. **(A)** Percentages of CD56^dim^ NK cells and CD56^bright^ NK cells among total PBMCs from HVs (*n*=12) and HCC patients (*n*=12). Data are presented as mean ± SD. **(B)** MSI values for ILT2, NKG2A, CD47, Siglec-7, DNAM-1, 2B4, NKp46 and CD16 expression on CD56^dim^ NK cells from HCC patients (*n*=12) and HVs (*n*=12). *p<0.05, **p<0.01, ***p<0.001, by the Mann–Whitney U-test.

To compare NK cells between HCC patients and healthy subjects, we enrolled age and sex-matched HCC patients and HVs (**Supplementary Table 5**). The frequency of CD56^dim^ NK cells was reduced in HCC patients compared with HVs, while the frequencies of CD56^bright^ NK cells were comparable (**Figure 2A**). We examined the expression levels of 35 surface markers on CD56^dim^ NK cells in HCC patients and HVs, and expressed the results in a heatmap (**Supplementary Figure 3**). The expression levels of inhibitory receptors ILT2, NKG2A, and CD47 on CD56^dim^ NK cells were significantly higher in HCC patients than in HVs, while the expression levels of activating receptors Siglec-7, DNAM-1 (CD226), and 2B4 (CD244) were significantly lower (**Figure 2B**). Of interest, although NKp46 was reported to be one of the major activating receptors of NK cells (11), the NKp46 expression levels were comparable between HCC patients and HVs (**Figure 2B**). And also CD16 expression levels were comparable between HCC patients and HVs (**Figure 2B**). The frequency of ILT2 on CD56^dim^ NK cells was also upregulated in HCC patients compared with HVs, while the frequencies of DNAM-1 and 2B4 were reduced (**Supplementary Figure 4A**). These results suggest that CD56^dim^ NK cells are reduced with enhanced expression of inhibitory receptors and reduced expression of activating receptors.

### Increase of ILT2 on intrahepatic CD56^dim^ NK cells in HCC patients

**Figure 3:**
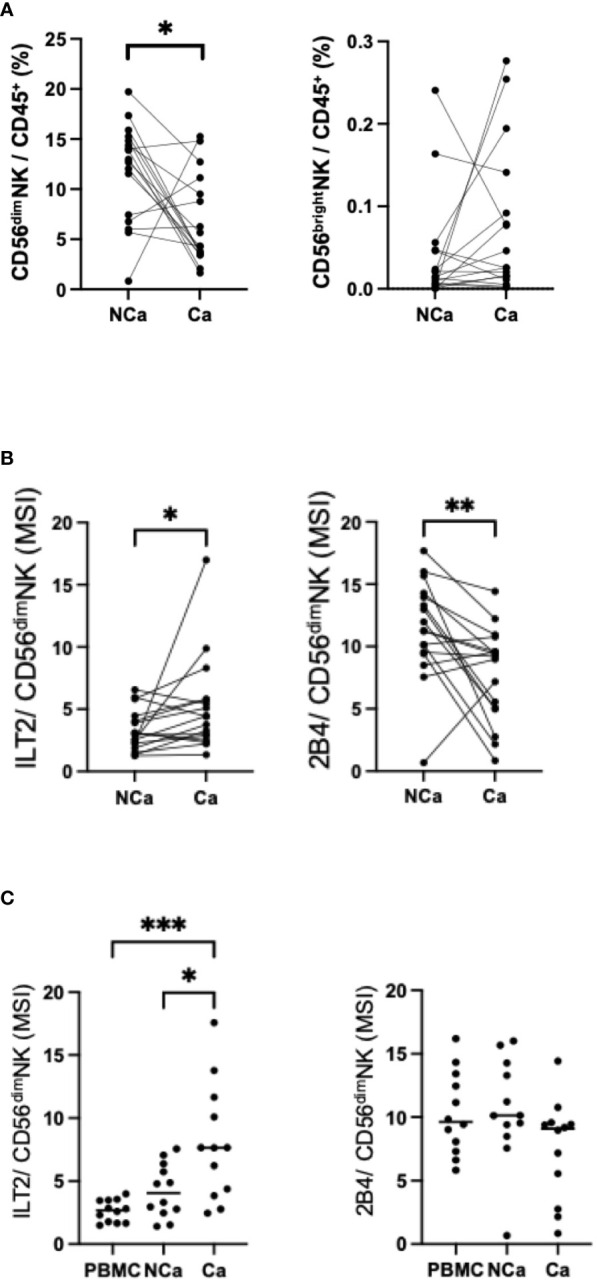
Changes in intrahepatic CD56^dim^ NK cells from HCC tissues. **(A)** Percentages of intrahepatic CD56^dim^ NK cells (CD45^+^CD3^−^CD14^−^CD56^dim^) and CD56^bright^ NK cells (CD45^+^CD3^−^CD14^−^CD56^bright^) from HCC tissue (Ca) and non-cancerous adjacent liver tissue (NCa) in 17 HCC patients. **(B)** MSI values for ILT2, PD-1, Tim-3, Siglec-9, Siglec-10, CD49a, NKp30, CXCR6, CX3CR1, HLA-DR, NKp44, CD57, and 2B4 expression on Ca-CD56^dim^ NK cells and NCa-CD56^dim^ NK cells. **(C)** MSI values and percentages of ILT2 expression on peripheral, intrahepatic NCa, and Ca CD56^dim^ NK cells. *p<0.05, **p<0.01, ***p<0.001, by the Friedman test with Dunn’s multiple comparison test.

Next, we compared the frequencies and surface markers of hepatic NK cells from HCC patients between paired cancerous (Ca) and non-cancerous (NCa) tissues (**Supplementary Table 1**). The frequency of Ca-CD56^dim^ NK cells was lower than the frequency of NCa-CD56^dim^ NK cells (6), while the frequencies of CD56^bright^ NK cells were comparable (**Figure 3A**). In cancerous tissue, the expression and frequency of ILT2 on CD56^dim^ NK cells were significantly higher, while the expression and frequency of 2B4 were lower, compared with their counterparts in non-cancerous tissue (**Figure 3B**, **Supplementary Figure 4B**). We further compared the expression of ILT2 on CD56^dim^ NK cells from HCC patients between the peripheral and intrahepatic compartments. Intrahepatic NK cells exhibited higher levels of ILT2 than peripheral NK cells, with higher expression in cancerous tissue than in non-cancerous tissue (**Figure 3C**). By contrast, the expression levels of 2B4 on CD56^dim^ NK cells did not differ between the two locations (**Figure 3C**). These results show that ILT2 on CD56^dim^ NK cells may be altered according to the age of patients and that its expression is higher in intrahepatic and HCC tissues than in peripheral tissue. Based on these observations, we focused on ILT2 as a signature molecule of NK cells in HCC patients.

### MIF and CXCR4 are involved in the ILT2 expression on CD56^dim^ NK cells

**Figure 4:**
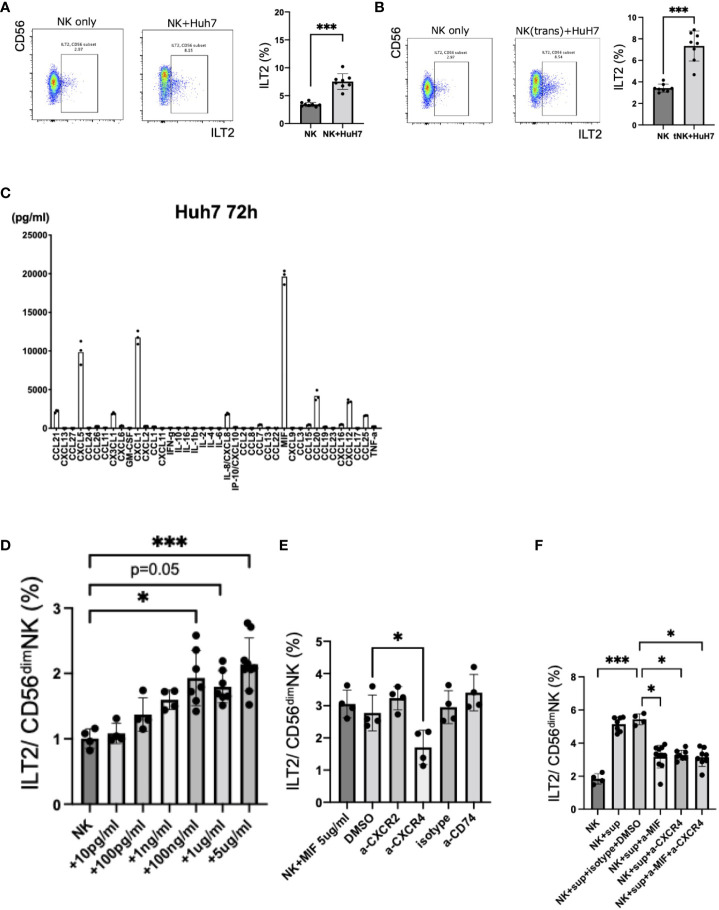
Induction of ILT2 expression on CD56^dim^ NK cells by HCC cell lines. **(A)** Representative flow cytometry plots showing the expression levels (%) of ILT2 on CD56^dim^ NK cells from NK cells only or NK cells co-cultured with Huh7 cells for 72 h with 1 ng/mL IL-15. ***p<0.001, by the Mann–Whitney U-test. **(B)** Representative flow cytometry plots showing the expression levels (%) of ILT2 on CD56^dim^ NK cells from NK cells only or NK cells co-cultured indirectly with Huh7 cells using transwell inserts for 72 h with 1 ng/mL IL-15. ***p<0.001, by the Mann–Whitney U-test. **(C)** Analysis of 40 chemokines and cytokines in culture supernatants of an HCC cell line (Huh7). **(D)** Induction of ILT2 expression by recombinant MIF. *p<0.05, ***p<0.001, by the Kruskal–Wallis test with Dunn’s multiple comparison test. **(E)** Inhibition of induction of ILT2 expression by recombinant MIF (5 μg/mL) for 48 h in the presence of CXCR2 antagonist, CXCR4 antagonist, anti-CD74 blocking antibody, DMSO, or isotype antibody. *p<0.05, by the Kruskal–Wallis test with Dunn’s multiple comparison test. **(F)** Inhibition of induction of ILT2 expression by Huh7 cell culture supernatants for 48 h in the presence of CXCR4 antagonist, anti-MIF blocking antibody, CXCR4 antagonist, anti-MIF blocking antibody, DMSO, and isotype antibody. ns, not significant.

To investigate the mechanisms of ILT2 induction on CD56^dim^ NK cells in HCC patients, we performed *in vitro* co-cultures of NK cells recovered from HVs and HCC cell lines. The expression of ILT2 on CD56^dim^ NK cells was increased in the presence of Huh7 cells (**Figure 4A**, **Supplementary Figure 5A**). The enhanced expression of ILT2 on CD56^dim^ NK cells was not reduced with transwell insert cultures, suggesting that soluble factors from Huh7 cells are involved in the induction of ILT2 (**Figure 4B**, **Supplementary Figure 5B**). In support of this notion, culture supernatants from Huh7 cells alone increased ILT2 expression on CD56^dim^ NK cells (**Supplementary Figure 5C**). To further explore this finding, we examined 40 chemokines and cytokines in culture supernatants of Huh7 cells with or without NK cells. We found that the concentrations of CCL21, CXCL5, CX3CL1, CXCL1, CXCL8, MIF, CCL20, CXCL12, and CCL25 were high in Huh7 cell supernatants (**Figure 4C**). Among these factors, MIF was the only factor that was also increased in the supernatants from other HCC cell lines (**Supplementary Figure 5D**). We compared the serum levels of the same factors between HCC patients and HVs. Again, the MIF level was significantly higher in HCC patients than in HVs (**Supplementary Table 6**).

Next, we cultured NK cells from HVs with various concentrations of recombinant MIF and examined the expression of ILT2. Recombinant MIF induced expression of ILT2 on CD56^dim^ NK cells in a dose-dependent manner (**Figure 4D**). It has been reported that CXCR4, CXCR2, and CD74 comprise a family of receptors for MIF (12). Because CD74 and CXCR4 were expressed on CD56^dim^ NK cells (**Supplementary Figure 5E**), we performed blocking experiments with masking antibodies against CXCR4 and CD74. The enhanced expression of ILT2 on CD56^dim^ NK cells in the presence of MIF was decreased after anti-CXCR4 antibody treatment (**Figure 4E**). Similar results were observed for anti-MIF and/or anti-CXCR4 antibodies in co-cultures of NK cells with Huh7 cell supernatants (**Figure 4F**). In contrast, antibodies against CXCR2 and CD74 failed to exert these effects (**Figure 4E**). Therefore, HCC-derived MIF and its receptor CXCR4 may be involved in the induction of ILT2 on CD56^dim^ NK cells.

### Impaired functions of ILT2^+^CD56^dim^ NK cells and their restoration by anti-ILT2 antibody treatment

**Figure 5:**
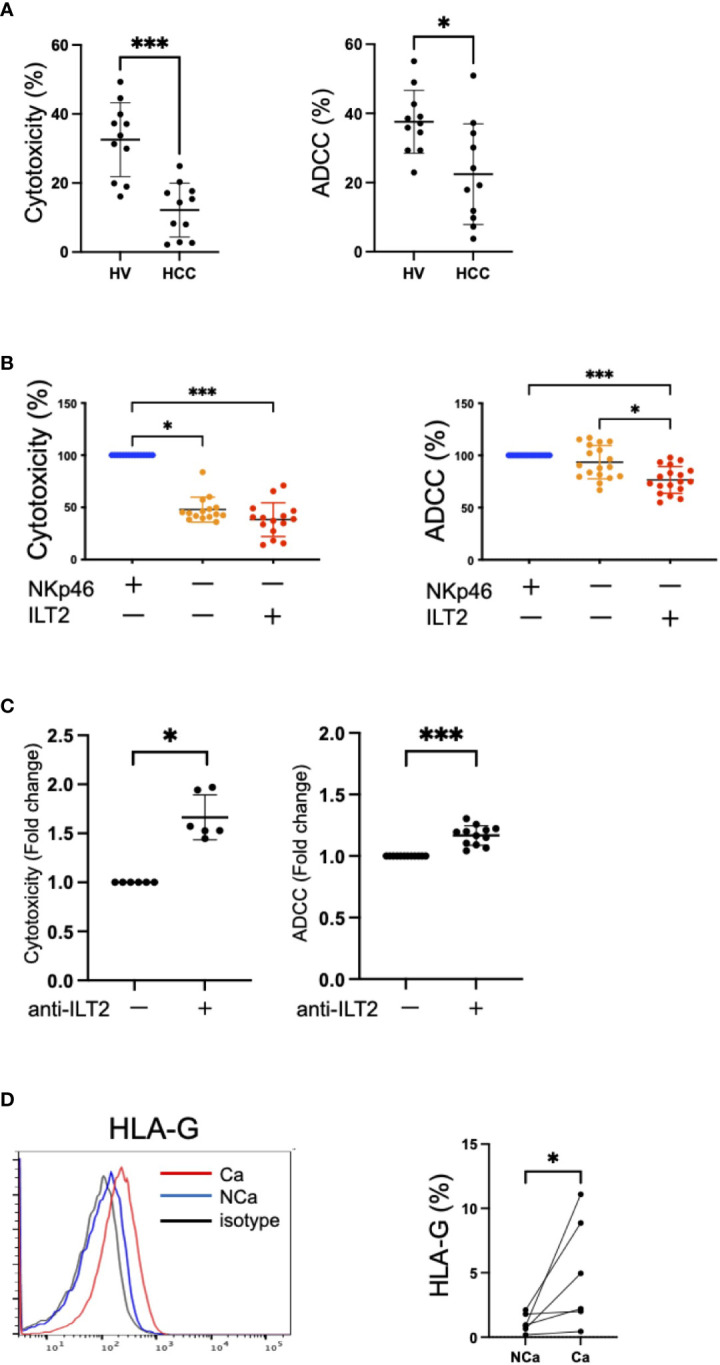
Dysfunction of ILT2^+^CD56^dim^ NK cells. **(A)** Cytotoxicity and ADCC assays of peripheral NK cells from HVs and HCC patients against K562 cells treated with 200 U/mL IL-2 or Daudi cells treated with 10 μg/mL rituximab were performed at 5:1 or 10:1 effector/target (E:T) ratios, respectively. *p<0.05, ***p<0.001, by the Mann–Whitney U-test. **(B)** NKp46^−^ILT2^+^, NKp46^+^ILT2^−^, or NKp46^−^ILT2^−^ subpopulations of CD56^dim^ NK cells were sorted by flow cytometry. Cytotoxicity and ADCC assays of these subpopulations against K562 cells treated with 200 U/mL IL-2 or Daudi cells treated with 10 μg/mL rituximab were performed at 2.5:1 E:T ratio. The red plots represent NKp46^−^ILT2^+^ cells, the blue plots represent NKp46^+^ILT2^−^ cells, and the yellow plots represent NKp46^−^ILT2^−^ cells. *p<0.05, ***p<0.001, by the Friedman test with Dunn’s multiple comparison test. **(C)** Functional recovery of ILT2^+^CD56^dim^ NK cells by anti-ILT2 blocking antibody treatment. The cytotoxicity and ADCC assays of ILT2^+^CD56^dim^ NK cells treated with anti-ILT2 blocking antibody. The cytotoxicity and ADCC capacities of ILT2^+^CD56^dim^ NK cells with anti-ILT2 blocking antibody treatment were evaluated by the fold changes in the presence or absence of the anti-ILT2 blocking antibody. *p<0.05, ***p<0.001, by the Mann–Whitney U-test. **(D)** Histograms showing the expression levels (%) of HLA-G on NCa and Ca liver tissue evaluated by flow cytometry. The red, blue, and black lines indicate Ca liver tissue, NCa liver tissue, or isotype, respectively. *p<0.05, by the Mann–Whitney U-test.

We compared the functions of NK cells recovered from HCC patients and HVs. The cytotoxicity and ADCC capacities of NK cells were impaired in HCC patients compared with HVs (**Figure 5A**). Next, we examined the functions of CD56^dim^ NK cells in terms of expression of ILT2 and NKp46. Because the expressions of ILT2 and NKp46 on CD56^dim^ NK cells are mutually exclusive, we compared the cytotoxicity and ADCC capacities among three cell subsets: ILT2^+^NKp46^−^, ILT2^−^NKp46^+^, and ILT2^−^NKp46^−^ cells. We found that ILT2^+^CD56^dim^ NK cells were functionally impaired compared with their ILT2^−^ counterparts regardless of NKp46 expression (**Figure 5B**).

To evaluate the possibility of ILT2 as a target for functional regulation of NK cells, we conducted cytotoxicity and ADCC assays of NK cells in the presence of a masking antibody against ILT2. Functional improvement of NK cells was observed after anti-ILT2 antibody treatment (**Figure 5C**). Furthermore, HLA-G, a ligand for ILT2, was more highly expressed in HCC tissue than in non-cancerous tissue (**Figure 5D**). These results show that ILT2 is a functional molecule of CD56^dim^ NK cells and may have potential as a possible target for reinvigoration of impaired CD56^dim^ NK cells in HCC patients.

## Discussion

NK cells are important effectors in cancer immune surveillance. In the present study, we found that CD56^dim^ NK cells are a major population in the IHLs in cancerous and non-cancerous tissues. Of interest is the finding that ILT2 is a signature molecule of CD56^dim^ NK cells with impaired killing capacity against tumor cells. While ILT2^+^ NK cells were not dysfunctional *per se*, the presence of its ligand on interacting cells was necessary to transmit inhibitory signals to NK cells. In this scenario, blocking of the cognitive binding between ILT2 and its ligand HLA-G restored the cytolytic ability of NK cells, thus raising the possibility that ILT2 and HLA-G may have potential as targets for immune modulation in the TME.

ILT2 is broadly expressed on NK cells, T cells, B cells, dendritic cells (DCs), and other immune cells, and it interacts with classical and non-classical MHC class I molecules, such as HLA-ABC, HLA-E, HLA-F, and HLA-G. In the present study, we found that HLA-G was more highly expressed in cancer tissues than in non-cancerous tissues. Enhanced expression of ILT2 on NK cells has been reported in various disease conditions, including cancers, autoimmune diseases, and chronic infections (13). In patients with triple-negative breast cancer or glioblastoma, ILT2 expression on NK cells was upregulated, and the degree of upregulation was correlated with the functional impairment against tumor cells (14, 15). The mechanisms for the ILT2 upregulation on NK cells remain to be clarified in the context of the tumor-bearing state. A previous study indicated that TGF-β may be responsible for the enhanced ILT2 expression on CD56^dim^CD16^+^ NK cells, which was associated with their dysfunction and susceptibility to apoptosis (16). In the present study, the expression of ILT2 on CD56^dim^ NK cells increased with closer localization to HCC in the liver, suggesting that factors with increasing or decreasing effects in the TME of the liver may be involved in the phenotypic change of NK cells.

MIF is an inflammatory cytokine and an important regulator of the innate immune response. In the search for humoral factors derived from HCC cells, we found that MIF, but not TGF-β, was one of the responsible factors for increasing the ILT2 expression on NK cells. Regarding receptors that interact with MIF, we found that CXCR4, but not CXCR2 or CD74, was involved by conducting blocking experiments *in vitro*. An immunomodulatory role of tumor-derived MIF has been reported previously. Specifically, overexpression of MIF contributed to tumor progression and the immune escape mechanism of HCC, ovarian carcinoma, and melanoma (17–19). More precisely, MIF downregulated the interaction between cancer-associated fibroblasts and immune cells including B cells, DCs, myeloid derived suppressor cells, monocytes, NK cells, and T cells via the CD74/CD44 and CXCR2/CXCR4 signaling pathways (19–21). In other studies related to HCC, the expression of MIF was higher in HCC tissues than in healthy or adjacent non-tumor liver tissues (17, 22). MIF was measurable in sera from patients with HCC, and the level was associated with hepatic tumor size and outcome of patients receiving transcatheter arterial chemo embolization (23). One of the limitations of the present study is the lack of analysis between serum MIF levels and expression levels of ILT2 on CD56^dim^ NK cells due to the scarcity of serum samples.

The role of NK cells as an immune effector against HCC has not been well established in clinical settings. Currently, reinvigoration of exhausted CD8^+^ T cells is one of the major approaches for the treatment of advanced HCC with ICIs. Considering that 20% of HCC patients show progressive disease under first-line atezolizumab and bevacizumab therapy, addition of an NK cell-mediated cytolytic effect may be a rational modality to overcome the primary resistance to ICIs. To this end, two options for NK cell treatment have been proposed: an adoptive transfer of activated autologous or allogeneic NK cells, and the use of antibodies against target cell-expressing molecules to promote ADCC. Several HCC-specific killer cell engagers have been reported as ADCC enhancers, such as antibodies targeting glypican-3 (24) and PD-L1 (25). In the present study, addition of an anti-ILT2 antibody improved the ADCC capacity *in vitro*, suggesting that targeting ILT2 or its ligand HLA-G as an engager may be an option for future investigations.

In summary, ILT2-positive CD56^dim^ NK cells have impaired cytolytic and ADCC capacities that are enhanced in cancerous tissues in HCC patients. ILT2 serves as a target for NK cell-mediated immune modulation, as shown by the functional improvement observed after anti-ILT2 antibody treatment *in vitro*. MIF and CXCR4 are associated with the induction of ILT2 on CD56^dim^ NK cells, the significance of which as a therapeutic target should be addressed in further studies.

## Data availability statement

The raw data supporting the conclusions of this article will be made available by the authors, without undue reservation.

## Ethics statement

The study protocol was approved by the ethics committees of the National Center for Global Health and Medicine (NCGM-A-000275-01) and the Cancer Institute Hospital of the Japanese Foundation for Cancer Research (2017-GA-1118). The studies were conducted in accordance with the local legislation and institutional requirements. The participants provided their written informed consent to participate in this study.

## Author contributions

TS: Writing – original draft, Writing – review & editing. SY: Writing – original draft, Writing – review & editing. TY: Writing – review & editing. TM: Writing – review & editing. EK: Writing – review & editing. YA: Writing – review & editing. NA: Writing – review & editing. TO: Writing – review & editing. TI: Writing – review & editing. HT: Writing – review & editing. TKaw: Writing – review & editing. YO: Writing – review & editing. YT: Writing – review & editing. AT: Writing – original draft, Writing – review & editing. TKan: Writing – original draft, Writing – review & editing.

## Glossary

**Table:**

ADCC	antibody-dependent cellular cytotoxicity
Ca	cancer
CCL	C-C motif chemokine ligand
CD	cluster differentiation
CFSE	carboxyfluorescein succinimidyl ester
CyTOF	cytometry by time-of-flight
CXCL	C-X-C motif chemokine ligand
CXCR	C-X-C motif chemokine receptor
CX3CL1	C-X3-C motif chemokine ligand 1
CX3CR1	C-X3-C motif chemokine receptor 1
DC	dendritic cell
DNAM-1	DNAX accessory molecule-1
FBS	fetal bovine serum
FCS	fetal calf serum
F stage	fibrosis stage
FcγRIIIa	Fc-gamma receptor IIIa
GM-CSF	granulocyte macrophage colony stimulating factor
HLA	human leukocyte antigen
HV	healthy volunteer
ICI	immune-checkpoint inhibitor
IFN-γ	interferon-gamma
IHL	intrahepatic lymphocyte
ILT2	immunoglobulin-like transcript 2
LAG-3	lymphocyte-activation gene 3
MIF	macrophage migration inhibitory factor
MSI	mean signal intensity
NCa	non-cancer
NK	natural killer
NKG2A	natural killer group 2 member A
NKG2D	natural killer group 2 member D
NKp30	natural killer cell p30
NKp44	natural killer cell p44
NKp46	natural killer cell p46
PBMC	peripheral blood mononuclear cell
PD-1	programmed cell death-1
PD-L1	programmed cell death-ligand 1
pNK	peripheral NK
Siglec	sialic acid-binding immunoglobulin-like lectin
TIGIT	T-cell immunoreceptor with immunoglobulin and ITIM domains
Tim-3	T-cell immunoglobulin and mucin domain 3
TME	tumor microenvironment
viSNE	visual stochastic network embedding.
