# Supplementary material for: Immunoglobulin-like transcript 2 as an impaired anti-tumor cytotoxicity marker of natural killer cells in patients with hepatocellular carcinoma
Source: Front Immunol. 2024 Apr 4;15:1389411. doi: 10.3389/fimmu.2024.1389411 (PMC11024250; doi:10.3389/fimmu.2024.1389411)
Supplement: Supplementary file 1 [file DataSheet_1.docx]

**Supplementary Tables**

**Supplementary Table 1.** **Clinicopathological characteristics of the HCC patients**

|  | HCC | | |  |
| --- | --- | --- | --- | --- |
|  | (n=17) | | |  |
| Age (years) | 70.0 | ± | 12.8 |  |
| Sex (male:female) | 14 : 3 | | |  |
| BMI (kg/m^2^) | 22.8 | ± | 3.2 |  |
| AST (IU/L) | 45.6 | ± | 32.0 |  |
| ALT (IU/L) | 40.9 | ± | 40.9 |  |
| T-bil (mg/dL) | 0.8 | ± | 0.3 |  |
| Alb (g/dL) | 4.1 | ± | 0.3 |  |
| Plt (x10^4^/µL) | 16.0 | ± | 6.1 |  |
| PT (%) | 103.6 | ± | 18.3 |  |
| Hb (g/dL) | 13.3 | ± | 1.2 |  |
| TG (mg/dL) | 130.0 | ± | 69.8 |  |
| T-Cho (g/dL) | 187.4 | ± | 52.1 |  |
| γGTP (IU/L) | 111.5 | ± | 134.8 |  |
| ALP (IU/L) | 372.1 | ± | 219.4 |  |
| HbA1c (%) | 6.1 | ± | 0.7 |  |
| Creatinine (mg/dL) | 0.80 | ± | 0.1 |  |
| Serum iron (μg/dL) | 92.3 | ± | 45.0 |  |
| Ferritin (ng/mL) | 285.2 | ± | 216.7 |  |
| Transferrin (mg/dL) | 255.9 | ± | 49.9 |  |
| TIBC (μg/dL) | 326.1 | ± | 59.3 |  |
| AFP (ng/mL) | 251.4 | ± | 860.9 |  |
| PIVKA-Ⅱ (mAU/mL) | 5314 | ± | 13147 |  |
| ALBI score | -2.7 | ± | 0.3 |  |
| Etiology (HCV/ HBV/ NBNC/ Alc) | 6/ 3/ 6/ 2 | | |  |
| F stage (0/ 1/ 2/ 3/ 4) | 2/ 0/ 4/ 5/ 6 | | |  |
| Maximal tumor size (cm) | 5.0 | ± | 3.0 |  |
| Histological differentiation grade  (Well/ Moderately/ Poor) | 1/ 14/ 2 | | |  |

Data are presented as patient number, mean ± SD, or score (range) as appropriate.

AFP, alpha-fetoprotein; Alb, serum albumin; ALBI, albumin-bilirubin grade; ALP, alkaline phosphatase; ALT, alanine aminotransferase; AST, aspartate aminotransferase; BMI, body mass index; F stage, fibrosis stage; Hb, hemoglobin; HbA1c, hemoglobin A1c; PIVKA-Ⅱ, protein induced by vitamin K absence or antagonist-Ⅱ; Plt, platelet count; PT, prothrombin time; T-bil, total bilirubin; T-cho, total cholesterol; TG, triglycerides; TIBC, total iron-binding capacity; TP, total protein; γGTP, gamma-glutamyl transpeptidase.

**Supplementary Table 2.** **Antibodies used for mass cytometry**

| Channel | Isotope | Marker | Function of Markers | Clone | Staining  Method | Catalog number |
| --- | --- | --- | --- | --- | --- | --- |
| 89 | Y | CD45 | lymphocytes identification | HI30 | surface | 3089003B |
| 106 | Cd | CD3 | T cell identification | UCHT1 | surface | Custom |
| 111 | Cd | CD38 | NK activation | HIT2 | surface | Custom |
| 112 | Cd | ILT2 (CD85j) | NK inhibitory | 292305 | surface | Custom |
| 113 | Cd | NKG2D (CD314) | NK activation | 149810 | surface | Custom |
| 114 | Cd | CD56 (NCAM) | NK identification | HCD56 | surface | Custom |
| 116 | Cd | TIGIT | NK inhibitory | 741182 | surface | Custom |
| 141 | Pr | CD27 | NK differentiation | O323 | surface | Custom |
| 143 | Nd | HLA-DR | APC identification | L243 | surface | 3143013B |
| 144 | Nd | CD69 | NK activation | FN50 | surface | 3144018B |
| 145 | Nd | KIR2DL1 | NK inhibitory | MAB1844 | surface | Custom |
| 146 | Nd | CXCR6 | NK differentiation | 56811 | surface | Custom |
| 147 | Sm | NKG2C (CD159c) | NK activation | MAB1381 | surface | Custom |
| 148 | Nd | Siglec-10 | Siglec family | AF2130 | surface | Custom |
| 149 | Sm | Siglec-9 | Siglec family | K8 | surface | Custom |
| 150 | Nd | CXCR1 | NK activation | 42705 | surface | Custom |
| 151 | Eu | CD96 | NK inhibitory | MAB6199 | surface | Custom |
| 152 | Sm | Siglec-7 (CD328) | Siglec family | 194211 | surface | 3152013B |
| 153 | Eu | TIM-3 | Inhibitory immune checkpoint | F38-2E2 | surface | 3153008B |
| 154 | Sm | CD49a | NK differentiation | AF5676 | surface | 3154016B |
| 155 | Gd | CX3CR1 | NK differentiation | AF5825 | surface | Custom |
| 156 | Gd | CD94 | NK inhibitory | 131412 | surface | Custom |
| 158 | Gd | CD200R | NK inhibitory | 380525 | surface | Custom |
| 159 | Tb | NKp30 (CD337) | NK activation | Z25 | surface | 3159017B |
| 160 | Gd | CD14 | Monocytes identification | M5E2 | surface | 3160001B |
| 162 | Dy | NKp46 (CD335) | NK activation | BAB281 | surface | Custom |
| 164 | Dy | TRAIL | NK activation | RIK-2 | surface | Custom |
| 165 | Ho | LAG-3 (CD223) | Inhibitory immune checkpoint | 11C3C65 | surface | 3165037B |
| 166 | Er | CD47 | NK inhibitory | 472603 | surface | 3166016B |
| 167 | Er | KIR3DL1 (CD158e1) | NK inhibitory | DX9 | surface | 3167013B |
| 168 | Er | CD160 | NK activation | 688327 | surface | Custom |
| 169 | Tm | NKG2A (159a) | NK inhibitory | Z199 | surface | 3169013B |
| 170 | Er | 2B4 (CD244) | NK activation | MAB10393 | surface | Custom |
| 171 | Yb | DNAM-1 (CD226) | NK activation | DX11 | surface | 3171013B |
| 172 | Yb | FasL | NK activation | NOK-1 | surface | Custom |
| 173 | Yb | KIR2DL2/L3 (CD158b) | NK inhibitory | DX27 | surface | 3173010B |
| 174 | Yb | PD-1 (CD279) | Inhibitory immune checkpoint | EH12.2H7 | surface | 3174020B |
| 175 | Lu | NKp44 (CD336) | NK activation | MAB22491 | surface | Custom |
| 176 | Yb | CD57 | NK differentiation | HCD57 | surface | 3176019B |
| 209 | Bi | CD16 | NK identification | 3G8 | surface | 3209002B |

CXCR6, C-X-C motif chemokine receptor 6; CX3CR1, C-X3-C motif chemokine receptor 1; DNAM-1, DNAX accessory molecule-1; HLA-DR, human leukocyte antigen-DR; ILT2, immunoglobulin-like transcript 2; KIR2DL1, killer cell immunoglobulin-like receptor 2DL1; KIR2DL2/L3, killer cell immunoglobulin-like receptor 2DL2/L3; KIR3DL1, killer cell immunoglobulin-like receptor, three Ig domains and long cytoplasmic tail 1; LAG-3, lymphocyte-activation gene 3; NKG2A, CD94/NK group 2 member A; NKG2D, CD94/NK group 2 family of C-type lectin-like receptors; NKp30, natural killer cell p30; NKp44, natural killer cell p44; NKp46, natural killer cell p46; PD-1, programmed cell death-1; Siglec, sialic acid-binding immunoglobulin-like lectin; TIGIT, T-cell immunoreceptor with Ig and ITIM domains; Tim-3, T-cell immunoglobulin and mucin domain 3.

**Supplementary Table 3.** **Antibodies used for flow cytometry**

| Antibody | Fluorophore | Clone | Vendor | Catalog number |
| --- | --- | --- | --- | --- |
| CD3 | APC-Cy7 | SK7 | Biolegend | 344818 |
| CD14 | APC-Cy7 | HCD14 | Biolegend | 325620 |
| CD16 | PE | 3G8 | BD Biosciences | 555407 |
| CD19 | APC-Cy7 | HIB19 | Biolegend | 302218 |
| CD56 | V450 | B159 | BD Biosciences | 560360 |
| HLA-G | PE | 87G | Abnova | MAB11223 |
| ILT2 | PE-Cy7 | GHI/75 | Biolegend | 333712 |
| NKp46 | APC | 9E2 | BD Biosciences | 558051 |

HLA-G, human leukocyte antigen-G; ILT2, immunoglobulin-like transcript 2; NKp46, natural killer cell p46.

**Supplementary Table 4. Correlations between surface marker expression levels (% or MSI) and age**

|  |  | Correlation of % | | | | |
| --- | --- | --- | --- | --- | --- | --- |
| Function of Markers | Marker | r | (95% CI) |  | p |  |
| NK differentiation | CD57 | 0.56 | (0.31 to 0.75) | | 0.0001 | *** |
|  | CD27 | -0.42 | (-0.65 to -0.12) | | 0.0058 | ** |
|  | CXCR6 | -0.24 | (-0.51 to 0.083) | | 0.1336 |  |
|  | CX3CR1 | 0.15 | (-0.17 to 0.44) | | 0.3330 |  |
|  | CD49a | 0.16 | (-0.16 to 0.45) | | 0.3155 |  |
| NK activation | Siglec-7 (CD328) | -0.56 | (-0.74 to -0.30) | | 0.0001 | *** |
|  | CD160 | -0.55 | (-0.73 to -0.28) | | 0.0002 | *** |
|  | NKp46 (CD335) | -0.49 | (-0.70 to -0.22) | | 0.0009 | *** |
|  | NKp30 (CD337) | -0.29 | (-0.55 to 0.024) | | 0.0618 |  |
|  | NKG2D (CD314) | -0.33 | (-0.59 to -0.024) | | 0.0307 | * |
|  | NKG2C (CD159c) | 0.12 | (-0.20 to 0.42) | | 0.4530 |  |
|  | 2B4 (CD244) | -0.019 | (-0.33 to 0.29) | | 0.9034 |  |
|  | DNAM-1 (CD226) | 0.038 | (-.28 to 0.35) | | 0.8114 |  |
|  | CD69 | 0.51 | (0.23 to 0.71) | | 0.0006 | *** |
|  | CD38 | -0.16 | (-0.45 to 0.16) | | 0.3258 |  |
|  | CXCR1 | -0.32 | (-0.57 to -0.0035) | | 0.0418 | * |
|  | HLA-DR | 0.78 | (0.62 to 0.88) | | <0.0001 | **** |
|  | NKp44 (CD336) | 0.19 | (-0.13 to 0.48) | | 0.2221 |  |
|  | FasL | 0.38 | (0.078 to 0.62) | | 0.0128 | * |
|  | TRAIL | 0.51 | (0.23 to 0.71) | | 0.0006 | *** |
| NK inhibitory | ILT2 (CD85j) | 0.50 | (0.34 to 0.76) | | <0.0001 | **** |
|  | PD-1 (CD279) | 0.26 | (-0.055 to 0.53) | | 0.0934 |  |
|  | TIM-3 | 0.27 | (-0.045 to 0.54) | | 0.0829 |  |
|  | LAG-3 (CD223) | 0.28 | (-0.037 to 0.54) | | 0.0744 |  |
|  | TIGIT | 0.25 | (-0.063 to 0.53) | | 0.1041 |  |
|  | CD96 | -0.22 | (-0.50 to 0.10) | | 0.1635 |  |
|  | NKG2A (159a) | -0.37 | (-0.69 to -0.19) | | 0.0015 | ** |
|  | CD94 | -0.23 | (-0.50 to 0.093) | | 0.1508 |  |
|  | Siglec-9 | 0.44 | (0.14 to 0.66) | | 0.0040 | ** |
|  | Siglec-10 | 0.73 | (0.55 to 0.85) | | <0.0001 | **** |
|  | KIR2DL1 | 0.13 | (-0.19 tp 0.43) | | 0.4078 |  |
|  | KIR2DL2/L3 | 0.22 | (-0.097 to 0.50) | | 0.1579 |  |
|  | KIR3DL1 | -0.31 | (-0.57 to 0.0012) | | 0.0448 | * |
|  | CD200R | 0.64 | (0.41 to 0.79) | | <0.0001 | **** |
|  | CD47 | -0.39 | (-0.62 to -0.085) | | 0.0114 | * |

|  |  | Correlation of MSI | | | | |
| --- | --- | --- | --- | --- | --- | --- |
| Function of Markers | Marker | r | (95% CI) |  | p |  |
| NK differentiation | CD57 | 0.44 | (0.15 to 0.66) | | 0.0037 | ** |
|  | CD27 | -0.31 | (-0.56 to 0.0074) | | 0.0490 | * |
|  | CXCR6 | 0.38 | (0.077 to 0.62) | | 0.0129 | * |
|  | CX3CR1 | 0.16 | (-0.16 to 0.45) | | 0.3144 |  |
|  | CD49a | -0.19 | (-0.47 to 0.13) | | 0.2324 |  |
| NK activation | Siglec-7 (CD328) | -0.57 | (0.76 to -0.34) | | <0.0001 | **** |
|  | CD160 | -0.55 | (0.74 to -0.29) | | 0.0001 | *** |
|  | NKp46 (CD335) | -0.51 | (-0.71 to -0.23) | | 0.0006 | *** |
|  | NKp30 (CD337) | -0.30 | (-0.56 to 0.017) | | 0.0566 |  |
|  | NKG2D (CD314) | -0.36 | (-0.61 to -0.057) | | 0.0183 | * |
|  | NKG2C (CD159c) | 0.11 | (-0.21 to 0.41) | | 0.4756 |  |
|  | 2B4 (CD244) | 0.061 | (-0.26 to 0.37) | | 0.6978 |  |
|  | DNAM-1 (CD226) | 0.37 | (0.062 to 0.61) | | 0.0168 | * |
|  | CD69 | 0.51 | (0.23 to 0.71) | | 0.0006 | *** |
|  | CD38 | -0.32 | (-0.58 to -0.013) | | 0.0362 | * |
|  | CXCR1 | -0.38 | (-0.62 to -0.074) | | 0.0138 | * |
|  | HLA-DR | 0.82 | (0.68 to 0.90) | | <0.0001 | *** |
|  | NKp44 (CD336) | 0.51 | (0.24 to 0.71) | | 0.0006 | *** |
|  | FasL | 0.39 | (0.093 to 0.63) | | 0.0099 | ** |
|  | TRAIL | 0.46 | (0.17 to 0.67) | | 0.0024 | ** |
| NK inhibitory | ILT2 (CD85j) | 0.57 | (0.32 to 0.75) | | <0.0001 | **** |
|  | PD-1 (CD279) | 0.53 | (0.26 to 0.72) | | 0.0003 | *** |
|  | TIM-3 | 0.17 | (-0.15 to 0.46) | | 0.2900 |  |
|  | LAG-3 (CD223) | 0.37 | (0.062 to 0.61) | | 0.0168 | * |
|  | TIGIT | 0.26 | (-0.060 to 0.53) | | 0.0998 |  |
|  | CD96 | -0.15 | (-0.44 to 0.17) | | 0.3353 |  |
|  | NKG2A (159a) | -0.34 | (-0.59 to -0.029) | | 0.0286 | * |
|  | CD94 | -0.27 | (-0.054 to 0.043) | | 0.0805 |  |
|  | Siglec-9 | 0.36 | (0.051 to 0.60) | | 0.0200 | * |
|  | Siglec-10 | 0.71 | (0.51 to 0.84) | | <0.0001 | **** |
|  | KIR2DL1 | -0.083 | (-0.39 to 0.24) | | 0.6025 |  |
|  | KIR2DL2/L3 | 0.2 | (-0,12 to 0.49) | | 0.1960 |  |
|  | KIR3DL1 | -0.34 | (-0.59 to -0.037) | | 0.0253 | * |
|  | CD200R | 0.49 | (0.2138 to 0.6980) | | 0.0009 | *** |
|  | CD47 | -0.58 | (-.75 to -0.32) | | <0.0001 | **** |

Spearman’s correlation rho (*r*) values and *p*-values are indicated.

**p*<0.05, ***p*<0.01, ****p*<0.001, *****p*<0.0001.

**Supplementary Table 5.** **Clinicopathological characteristics of the HVs and HCC patients**

|  | HV | | | | | | HCC | | | *P* value |
| --- | --- | --- | --- | --- | --- | --- | --- | --- | --- | --- |
|  | All (n=42) | | | Matched (n=12) | | | (n=12) | | |  |
| Age (years) | 52.0 | ± | 20.6 | 72.9 | ± | 9.4 | 73.1 | ± | 9.6 | NS |
| Sex (male:female) | 21 : 21 | | | 9 : 3 | | | 9 : 3 | | | NS |
| BMI (kg/m^2^) |  |  |  | 23.1 | ± | 2.3 | 21.9 | ± | 2.3 | NS |
| **AST (IU/L)** |  |  |  | 22.1 | ± | 4.6 | 50.3 | ± | 35.4 | **< 0.05** |
| **ALT (IU/L)** |  |  |  | 19.3 | ± | 6.8 | 41.1 | ± | 47.8 | **< 0.05** |
| T-bil (mg/dL) |  |  |  |  | - |  | 0.8 | ± | 0.2 |  |
| Alb (g/dL) |  |  |  |  | - |  | 4.0 | ± | 0.4 |  |
| **Plt (x10^4^/µL)** |  |  |  | 22.9 | ± | 3.7 | 15.2 | ± | 5.1 | **< 0.001** |
| PT (%) |  |  |  |  | - |  | 105.3 | ± | 20.8 |  |
| Hb (g/dL) |  |  |  |  | - |  | 13.0 | ± | 1.1 |  |
| TG (mg/dL) |  |  |  |  | - |  | 119.8 | ± | 58.4 |  |
| T-Cho (g/dL) |  |  |  |  | - |  | 181.8 | ± | 55.2 |  |
| γGTP (IU/L) |  |  |  |  | - |  | 127.2 | ± | 158.6 |  |
| ALP (IU/L) |  |  |  |  | - |  | 412.4 | ± | 242.2 |  |
| HbA1c (%) |  |  |  |  | - |  | 6.0 | ± | 0.5 |  |
| Serum creatinine (mg/dL) |  |  |  |  | - |  | 0.80 | ± | 0.2 |  |
| Serum iron (μg/dL) |  |  |  |  | - |  | 88.5 | ± | 51.0 |  |
| Serum ferritin (ng/mL) |  |  |  |  | - |  | 245.6 | ± | 128.3 | (n=11) |
| Serum transferrin (mg/dL) |  |  |  |  | - |  | 259.1 | ± | 57.8 | (n=11) |
| TIBC (μg/dL) |  |  |  |  | - |  | 328.5 | ± | 67.8 | (n=11) |
| AFP (ng/mL) |  |  |  |  | - |  | 333.7 | ± | 1026 |  |
| PIVKA-Ⅱ  (mAU/mL) |  |  |  |  | - |  | 7308 | ± | 15382 |  |
| ALBI score |  |  |  |  | - |  | -2.7 | ± | 0.3 |  |
| Etiology  (HCV/ NBNC/ Alc) |  |  |  |  | - |  | 6/ 5/ 1 | | |  |
| F stage (0/ 1/ 2/ 3/ 4) |  |  |  |  | - |  | 1/ 0/ 3/ 5/ 3 | | |  |
| Maximal tumor size (cm) |  |  |  |  | - |  | 5.2 | ± | 2.3 |  |
| Histological differentiation grade  (Well/ Moderately/ Poor) |  |  |  |  | - |  | 1/ 9/ 2 | | |  |

Data are presented as patient number, mean ± SD, or score (range) as appropriate.

The *p*-values were determined by the Mann–Whitney U-test.

AFP, alpha-fetoprotein; Alb, serum albumin; ALBI, albumin-bilirubin grade; ALP, alkaline phosphatase; ALT, alanine aminotransferase; AST, aspartate aminotransferase; BMI, body mass index; F stage, fibrosis stage; Hb, hemoglobin; HbA1c, hemoglobin A1c; HV, healthy volunteer; NS, not significant; PIVKA-Ⅱ, protein induced by vitamin K absence or antagonist-Ⅱ; Plt, platelet count; PT, prothrombin time; T-bil, total bilirubin; T-cho, total cholesterol; TG, triglycerides; TIBC, total iron-binding capacity; TP, total protein; γGTP, gamma-glutamyl transpeptidase.

**Supplementary Table 6. Detection of 40 chemokines and cytokines in sera from the HCC patients and HVs**

|  |  |  | HV |  | HCC |  |
| --- | --- | --- | --- | --- | --- | --- |
|  |  |  | (n=8) |  | (n=28) | *P* value |
| TNF-α | (pg/ml) |  | 26.4 ± 3.5 |  | 16.5 ± 6.1 | **** |
| MIF | (pg/ml) |  | 1013 ± 186.5 |  | 21824 ± 30660 | **** |
| IL-6 | (pg/ml) |  | 6.7 ± 2.8 |  | not detected |  |
| IL-4 | (pg/ml) |  | 33.5 ± 5.5 |  | 80.9 ± 20.8 | **** |
| IL-2 | (pg/ml) |  | 21.6 ± 4.1 |  | 3.0 ± 1.5 | * |
| IL-1b | (pg/ml) |  | 2.6 ± 0.5 |  | 3.6 ± 0.8 | *** |
| IL-16 | (pg/ml) |  | 124.2 ± 42.7 |  | 85.4 ± 77.4 | ** |
| IL-10 | (pg/ml) |  | 7.9 ± 2.9 |  | 3.8 ± 4.6 | *** |
| IFN-γ | (pg/ml) |  | 11.4 ± 4.4 |  | 8.2 ± 2.8 | * |
| GM-CSF | (pg/ml) |  | 11.6 ± 3.4 |  | 7.7 ± 6.9 | ** |
| CXCL9 | (pg/ml) |  | 27.5 ± 5.3 |  | 78.4 ± 107.0 |  |
| CXCL8 | (pg/ml) |  | 21.3 ± 25.6 |  | 31.77 ± 33.7 |  |
| CXCL6 | (pg/ml) |  | 38.3 ± 10.5 |  | 25.9 ± 18.3 | * |
| CXCL5 | (pg/ml) |  | 306.3 ± 152.7 |  | 275.3 ± 269.4 |  |
| CXCL2 | (pg/ml) |  | 467.8 ± 135.9 |  | 208.1 ± 165.3 | *** |
| CXCL16 | (pg/ml) |  | 381.5 ± 138.4 |  | 457.0 ± 167.7 |  |
| CXCL13 | (pg/ml) |  | 18.69 ± 6.0 |  | 34.9 ± 30.7 |  |
| CXCL12 | (pg/ml) |  | 473.6 ± 89.5 |  | 1659 ± 352.9 | **** |
| CXCL11 | (pg/ml) |  | 15.7 ± 7.0 |  | 32.7 ± 31.2 |  |
| CXCL10 | (pg/ml) |  | 110.9 ± 11.9 |  | 223.5 ± 241.9 |  |
| CXCL1 | (pg/ml) |  | 262.4 ± 53.9 |  | 65.8 ± 60.5 | * |
| CX3CL1 | (pg/ml) |  | 127.6 ± 36.7 |  | not detected |  |
| CCL8 | (pg/ml) |  | 67.7 ± 23.3 |  | 42.9 ± 18.5 | ** |
| CCL7 | (pg/ml) |  | 47.4 ± 7.4 |  | not detected |  |
| CCL3 | (pg/ml) |  | 25.79 ± 46.1 |  | 5.3 ± 2.9 |  |
| CCL27 | (pg/ml) |  | 829.2 ± 231.7 |  | 1236 ± 578.8 | * |
| CCL26 | (pg/ml) |  | 44.4 ± 10.4 |  | 12.1 ± 12.8 | **** |
| CCL25 | (pg/ml) |  | 611.3 ± 67.7 |  | 247.6 ± 144.5 | **** |
| CCL24 | (pg/ml) |  | 223.4 ± 84.2 |  | 204.8 ± 97.8 |  |
| CCL23 | (pg/ml) |  | 229.2 ± 127.8 |  | 199.4 ± 127.3 | * |
| CCL22 | (pg/ml) |  | 539.8 ± 143.4 |  | 501.4 ± 213.5 |  |
| CCL21 | (pg/ml) |  | 14087 ± 2143 |  | 19552 ± 4611 | ** |
| CCL20 | (pg/ml) |  | 3.0 ± 0.8 |  | 77.9 ± 71.3 | *** |
| CCL2 | (pg/ml) |  | 127.6 ± 35.1 |  | 47.2 ± 11.7 | **** |
| CCL19 | (pg/ml) |  | 95.1 ± 13.8 |  | 105.5 ± 109.6 |  |
| CCL17 | (pg/ml) |  | 139.2 ± 88.4 |  | 107.8 ± 97.5 |  |
| CCL15 | (pg/ml) |  | 4211 ± 1101 |  | 13676 ± 49126 |  |
| CCL13 | (pg/ml) |  | 105.3 ± 37.9 |  | 78.2 ± 40.8 |  |
| CCL11 | (pg/ml) |  | 46.5 ± 10.2 |  | 46.2 ± 14.8 |  |
| CCL1 | (pg/ml) |  | 46.4 ± 2.5 |  | 17.0 ± 7.5 | *** |

The *p*-values were determined by the Mann–Whitney U-test.

**p*<0.05, ***p*<0.01, ****p*<0.001, *****p*<0.0001.

MIF, macrophage migration inhibitory factor; IFN-γ, interferon-gamma; GM-CSF, granulocyte macrophage colony stimulating factor; CXCL, C-X-C motif chemokine ligand; CX3CL1, C-X3-C motif chemokine ligand 1; CCL, C-C motif chemokine ligand.

**Supplementary Figures**

**Figure S1. Expression of HLA-G on target cells.** Histograms showing the expression of HLA-G on K562 and Daudi cells as target cells for cytotoxicity and ADCC assays, respectively. The yellow and black lines indicate target cells or isotype cells, respectively.

**
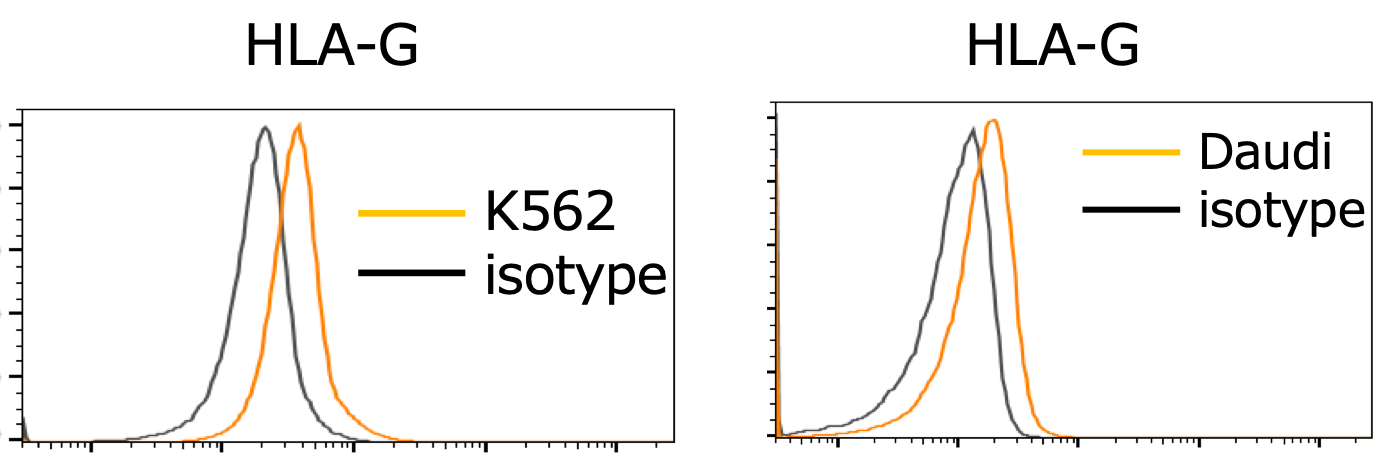
**

**Figure S2.** **Representative viSNE plots of peripheral CD56^dim^ NK cells from younger and older HVs.** The panels show the expression of the indicated surface proteins: CD57, Siglec-7, CD160, NKp46, PD-1, LAG-3, Siglec-10, ILT2, KIR2DL2/L3, and TIGIT. The color scale indicates a gradient from high (red) to low (blue) expression of the relevant protein. The red circles with dashed and solid lines indicate the CD56^dim^ NK subpopulation that was specifically detected in the older individual and not in the younger individual.


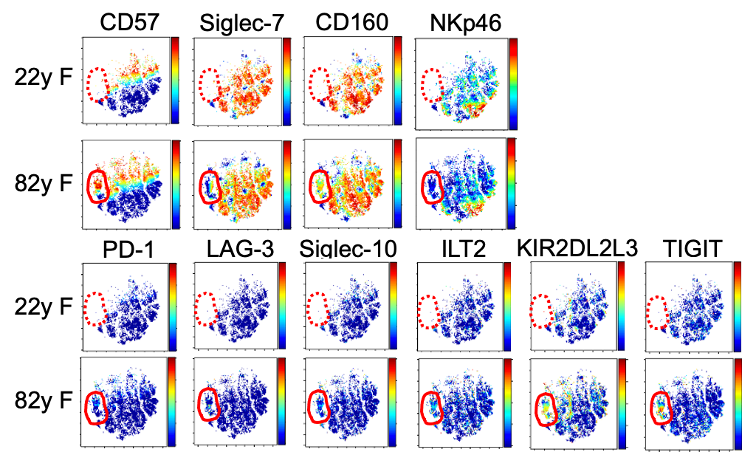


**Figure S3. Heatmap of surface marker expression on peripheral CD56^dim^ NK cells from HCC patients and HVs.** The horizontal axis represents 35 surface markers on CD56^dim^ NK cells. The vertical axis represents the samples from HCC patients and HVs. The red region represents upregulated markers and the blue region represents downregulated markers. The red circles indicate surface markers that were highly expressed on CD56^dim^ NK cells from HVs compared with HCC patients (Siglec-7, DNAM-1, 2B4). The blue circles indicate surface markers that were highly expressed on CD56^dim^ NK cells from HCC patients compared with HVs (NKG2A, ILT2, CD47).

**
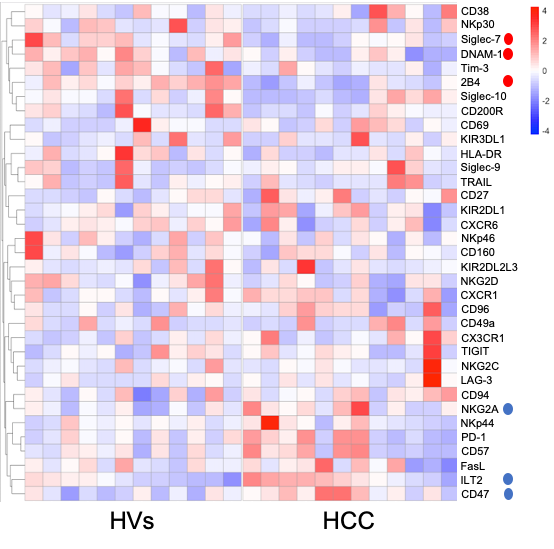
**

**Figure S4. Change in NK cells in HCC patients.** (A) Frequencies (%) of ILT2, DNAM-1, and 2B4 expression on CD56^dim^ NK cells from HCC patients (*n*=12) and HVs (*n*=12). Data are presented as individual values with a mean line. ***p*<0.01, ****p*<0.001, by the Mann–Whitney U-test. (B) Frequencies (%) of ILT2 and 2B4 expression on Ca-CD56^dim^ NK cells and NCa-CD56^dim^ NK cells. **p*<0.05 by a paired *t*-test.

**A**

**
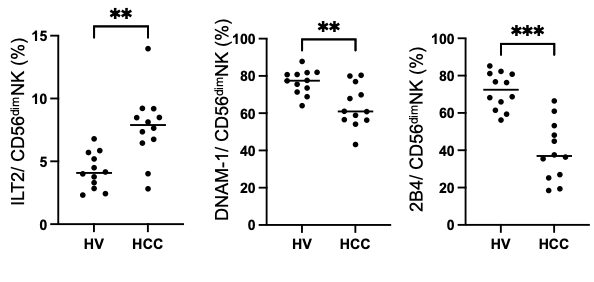
**

**B**

**
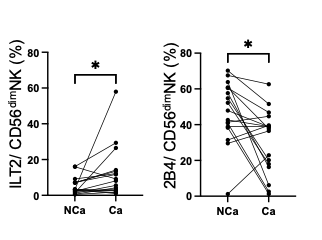
**

**Figure S5. Induction of ILT2 on CD56^dim^ NK cells.** (A) Expression levels (%) of ILT2 on CD56^dim^ NK cells from NK cells only or NK cells cocultured with PLC-PRF5 or HLE cells for 72 h with 1 ng/mL IL-15. ***p*<0.01, by the Mann–Whitney U-test. (B) Expression levels (%) of ILT2 on CD56^dim^ NK cells from NK cells only or NK cells cocultured indirectly with PLC-PRF5 or HLE cells using transwell inserts for 72 h with 1 ng/mL IL-15. **p*<0.05, by the Mann–Whitney U-test. (C) Induction of ILT2 expression by culture supernatants of Huh7 cells. ***p*<0.01, by the Mann–Whitney U-test. (D) Detection of 40 chemokines and cytokines in culture supernatants of HCC cells (PLC-PRF5 and HLE). (E) Expression levels of CD74, CXCR4, and CXCR2 as receptors of MIF on CD56^dim^ NK cells determined by flow cytometry. Representative dot plots and histograms showing CXCR2^+^, CXCR4^+^, and CD74^+^ cells among CD56^dim^ NK cells are presented.

**A**

**
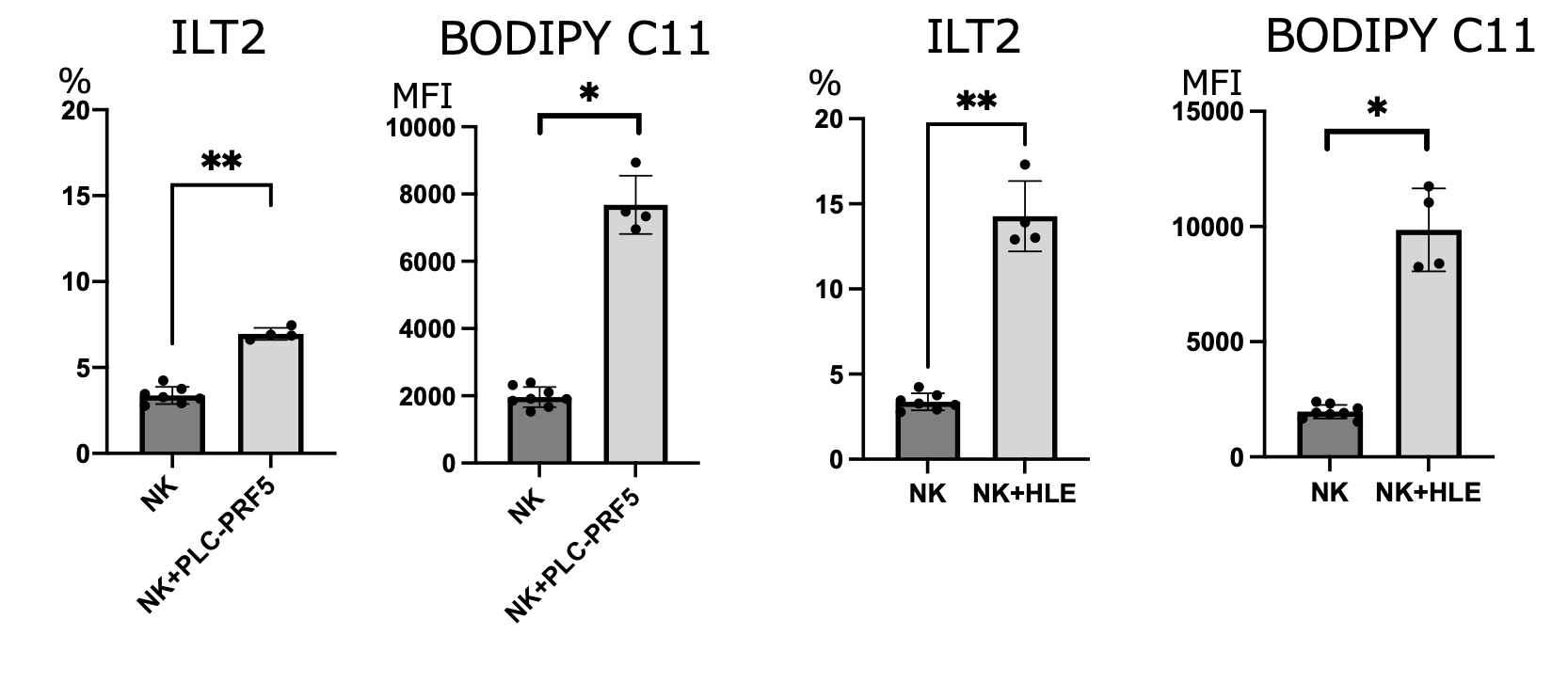

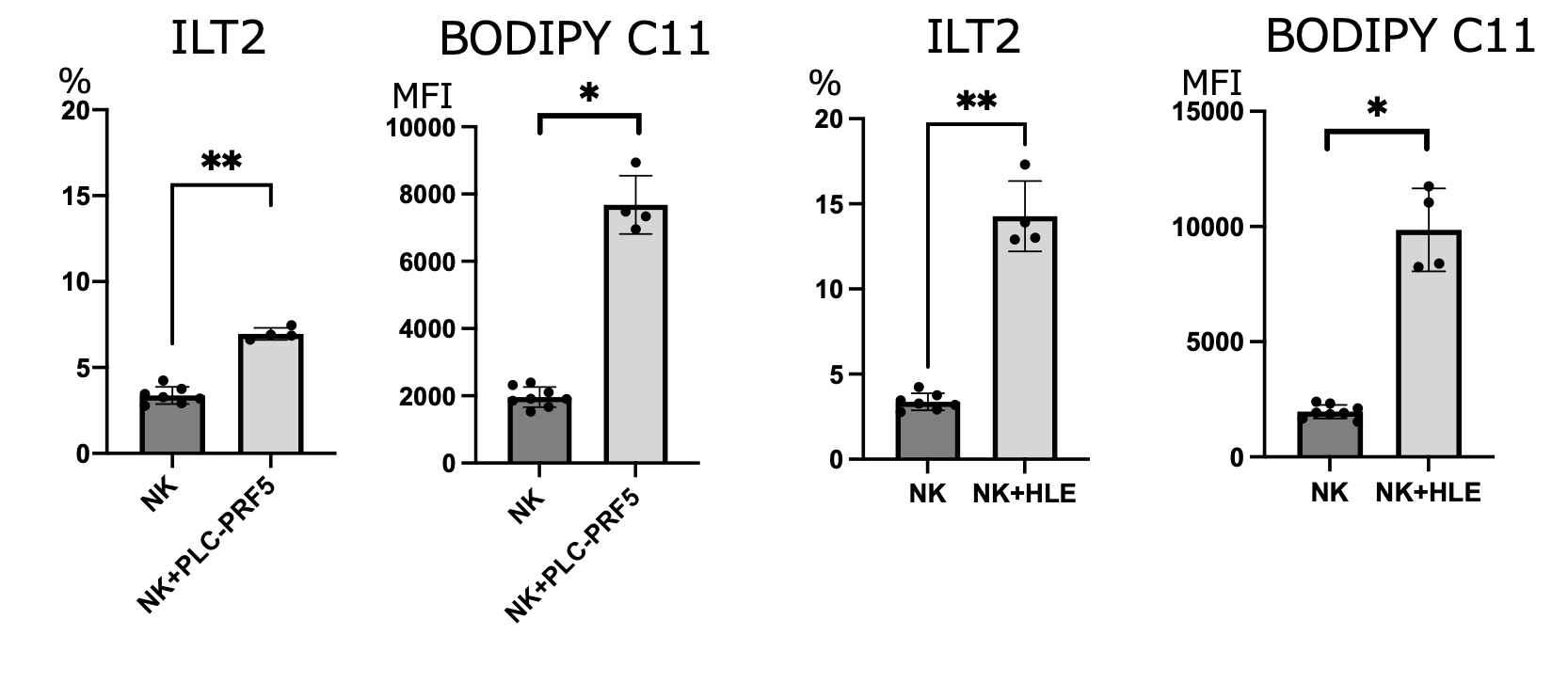
**

**B**

**
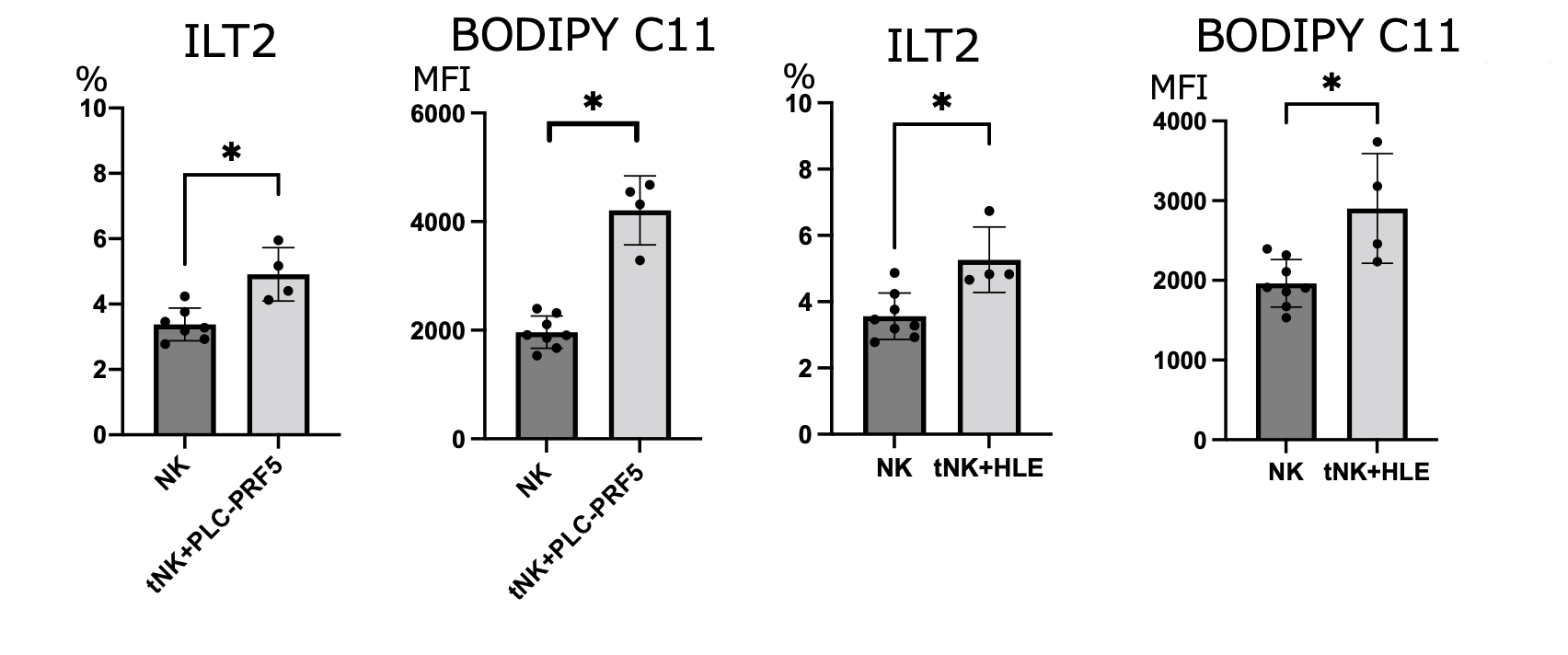

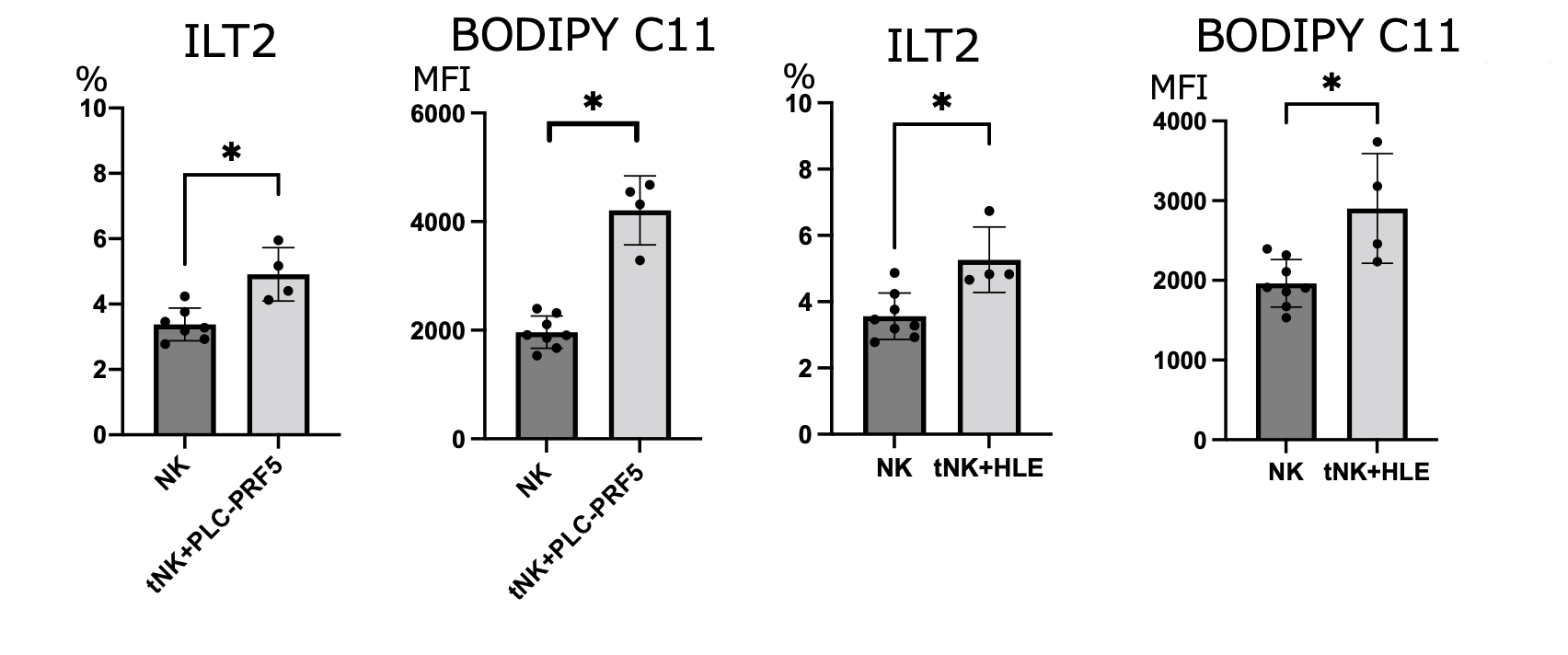
**

**C**


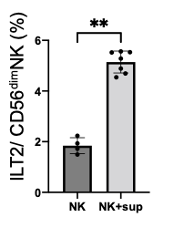


**D**

**
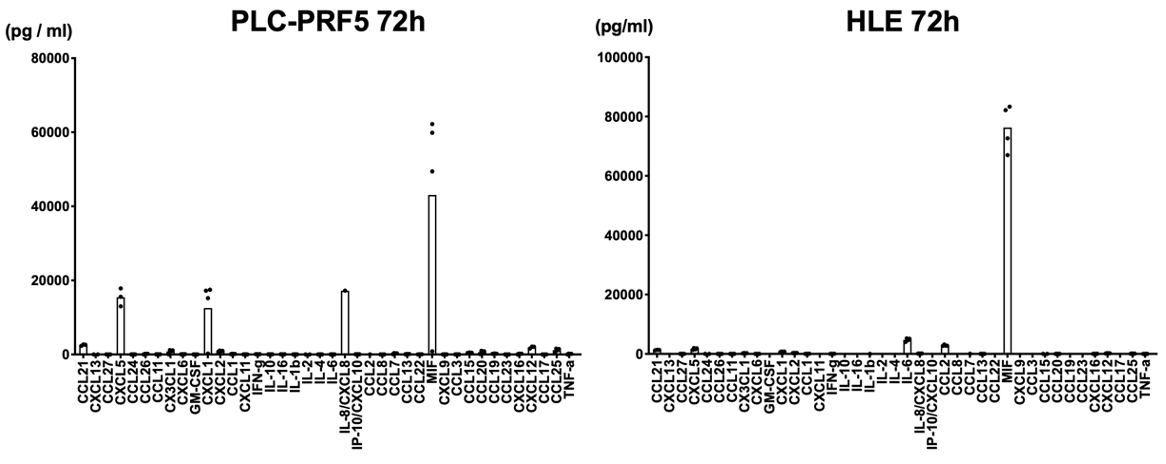
**

**E**

**
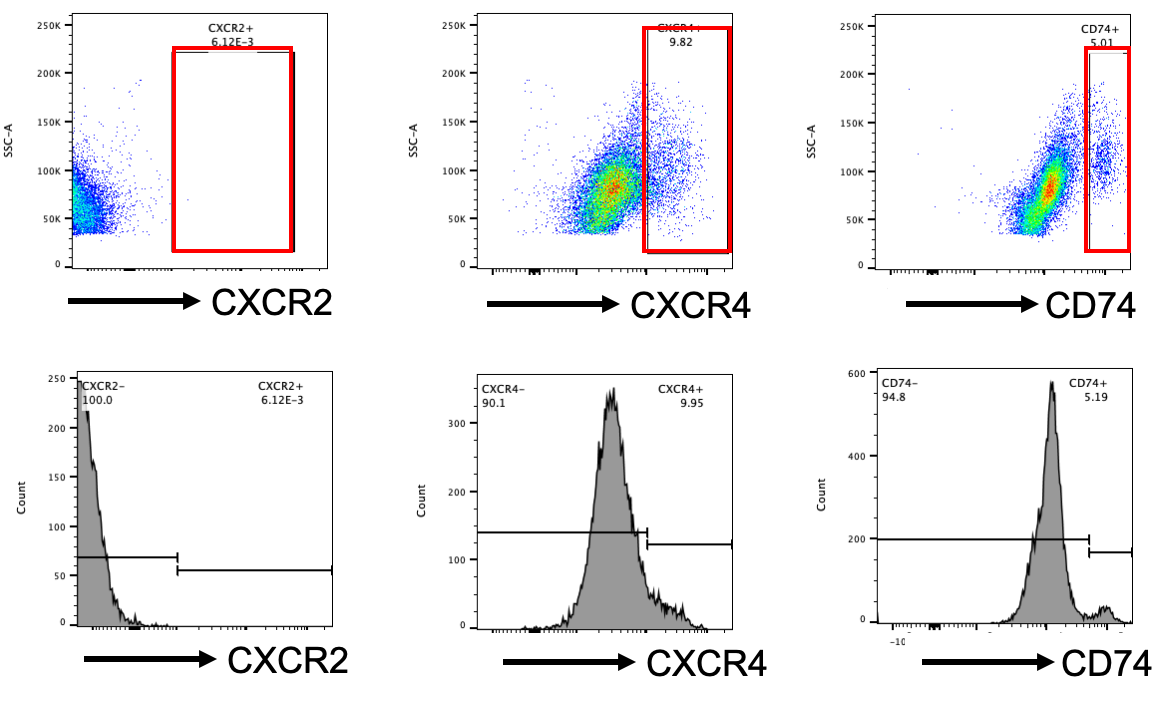
**
